# Exceptional Complex Chromosomal Rearrangements in Three Generations

**DOI:** 10.1155/2015/321014

**Published:** 2015-02-03

**Authors:** Hannie Kartapradja, Nanis Sacharina Marzuki, Mark D. Pertile, David Francis, Lita Putri Suciati, Helena Woro Anggaratri, Debby Dwi Ambarwati, Firman Prathama Idris, Harry Lesmana, Hidayat Trimarsanto, Chrysantine Paramayuda, Alida Roswita Harahap

**Affiliations:** ^1^Eijkman Institute for Molecular Biology, Jl. Diponegoro 69, Jakarta 10430, Indonesia; ^2^Victorian Clinical Genetics Services (VCGS), Royal Children's Hospital, Flemington Road, Melbourne, VIC 3052, Australia; ^3^Agency for the Assessment and Application of Technology, Jl. MH Thamrin 8, Jakarta 10340, Indonesia

## Abstract

We report an exceptional complex chromosomal rearrangement (CCR) found in three individuals in a family that involves 4 chromosomes with 5 breakpoints. The CCR was ascertained in a phenotypically abnormal newborn with additional chromosomal material on the short arm of chromosome 4. Maternal karyotyping indicated that the mother carried an apparently balanced CCR involving chromosomes 4, 6, 11, and 18. Maternal transmission of the derivative chromosome 4 resulted in partial trisomy for chromosomes 6q and 18q and a partial monosomy of chromosome 4p in the proband. Further family studies found that the maternal grandmother carried the same apparently balanced CCR as the proband's mother, which was confirmed using the whole chromosome painting (WCP) FISH. High resolution whole genome microarray analysis of DNA from the proband's mother found no evidence for copy number imbalance in the vicinity of the CCR translocation breakpoints, or elsewhere in the genome, providing evidence that the mother's and grandmother's CCRs were balanced at a molecular level. This structural rearrangement can be categorized as an exceptional CCR due to its complexity and is a rare example of an exceptional CCR being transmitted in balanced and/or unbalanced form across three generations.

## 1. Introduction

Constitutional complex chromosomal rearrangements (CCRs) usually involve at least two chromosomes and three breakpoints with varied outcomes (simple or 3-break insertions are excluded) [1–4]. These abnormalities may involve distal segments causing reciprocal translocation, or interstitial segments leading to insertion, inversion, deletion, or duplication, or they may involve a combination of both distal and interstitial segments [1, 3]. One chromosome may also have more than one aberration such as an inversion and a translocation that can coexist on the same chromosome [1].

The phenotype of the CCR carrier varies from normal to abnormal with congenital abnormalities and/or intellectual disability. The likelihood of an abnormal phenotype increases with the number of breakpoints associated with the de novo, apparently balanced CCR (BCCR) [5–7]. Approximately 255 cases of CCRs involving three or more chromosomes have been published. Cases involving 4 chromosomes with 5 breakpoints are classified as exceptional and can be highly complex in nature [4]. The risk of spontaneous abortion in a pregnancy from a CCR carrier can be as high as 50 to 100% [5], whereas 18% of all live births to CCR carriers result in phenotypically abnormal offspring [8]. According to Gardner and Sutherland [1], CCRs can be classified into three groups based on the number of breakpoints and type of arrangement. These are the following. (1)* Three-way exchange with three breaks from three chromosomes*: most three-way CCRs are familial and are usually transmitted through the mother. They are the most common type of CCRs. (2)* Double two-way exchange with a coincidence of two separate simple reciprocal translocations*: the double two-way CCR is not a true CCR and might be described as a double or a multiple rearrangement. (3)* Exceptional CCRs with more complicated rearrangements*: most exceptional CCRs are de novo rearrangements and they are more commonly associated with abnormal phenotype.

Most familial transmissions of CCRs are through the mother [2, 6]. Phenotypically normal female BCCR carriers are usually ascertained following investigation for recurrent abortions or after the birth of an abnormal child. In contrast, phenotypically normal BCCR males are more frequently ascertained following investigation for infertility [6, 8–10]. Here we report a very rare familial exceptional CCR in 3 generations which includes two normal phenotype BCCR individuals.

## 2. Case Presentation

**Figure 1:**
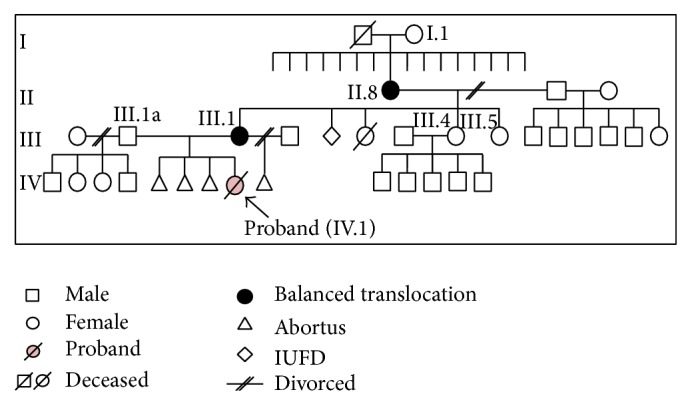
Pedigree of the family.

The proband was a one-day-old female referred to our clinic for chromosome analysis. She had multiple congenital anomalies with facial dysmorphism, cleft lip, micrognathia, and intrauterine growth retardation (IUGR). Her weight was 1630 grams and length 38 cm. The proband (individual IV.1) was delivered from an uneventful, full term pregnancy of a 30-year-old mother with an obstetrical status of G_5_P_1_A_4_ from 2 marriages. The four previous pregnancies ended in abortions at around 20 weeks of gestation. The maternal grandmother of the proband (individual II.8) was the 8th of 16 normal phenotype siblings. Her 4th child, who died at 8 months old without any specific causes, was born prematurely with normal phenotype. The great grandmother from the mother's lineage (individual I.1) has 16 children with no miscarriage history. The proband's aunt (individual III.4) has 5 phenotypically normal sons and another aunt (individual III.5) was not involved in this study (Figure 1). Patient histories were negative for radiation exposure and drug consumption during pregnancy.

**Figure 2:**
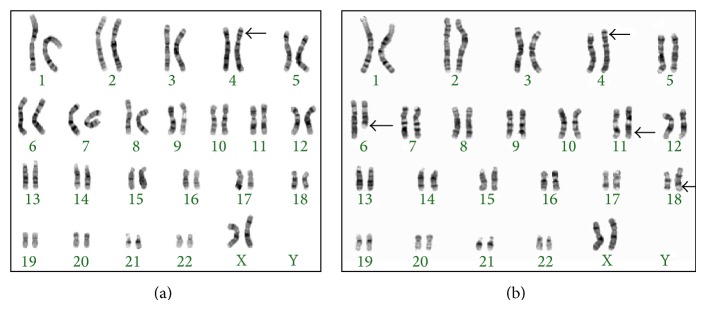
Karyotyping result: (a) proband and (b) mother and grandmother.

Cytogenetic investigations were carried out on 20 metaphase cells of phytohaemagglutinin- (PHA-) stimulated peripheral blood cultures using standard procedures, and high resolution GTL banding was performed. Analysis undertaken on metaphase chromosomes from the proband at the 550-band level according to ISCN 2009 [11] showed the unbalanced karyotype: 46,XX,der(4)(18qter→q21.3::6q13q21::4p14→qter)mat (Figure 2(a)). This result was determined after analysis of the mother's karyotype showed a BCCR with karyotype 46,XX,der(4)(18qter→q21.3::6q13q21::4p14→qter),der(6)t(4;6)(p14;q21),der(11)t(6;11)(q21;q21),der(18)t(11; 18)(q21;q21.3) (Figure 2(b)). We extended our chromosome analysis to other family members and found the maternal grandmother (individual II.8) had the same BCCR as the proband's mother. The great grandmother (I.1) and another aunt of the proband (individual III.4) had normal karyotypes, which suggested that the BCCR arose as a de novo event in the proband's grandmother. This conclusion is based on the premise that the deceased great grandfather is very unlikely to have carried the BCCR, as he fathered 16 normal phenotype children in the absence of miscarriage.

**Figure 3:**
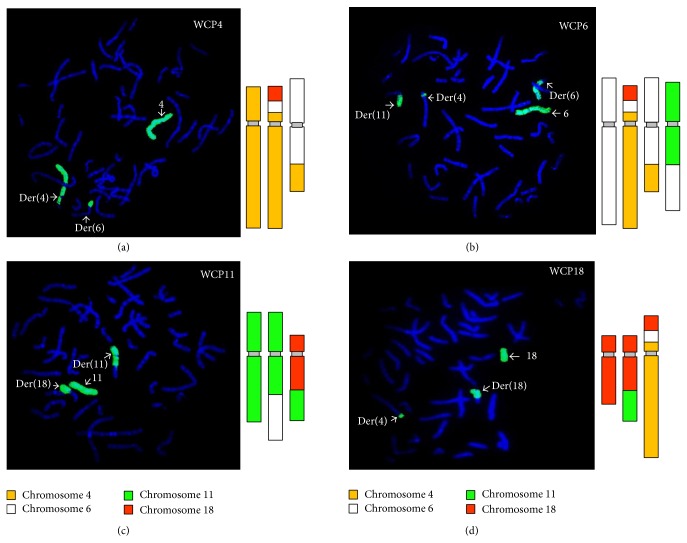
Whole chromosome painting FISH: (a) chromosome 4, (b) chromosome 6, (c) chromosome 11, and (d) chromosome 18.

**Figure 4:**
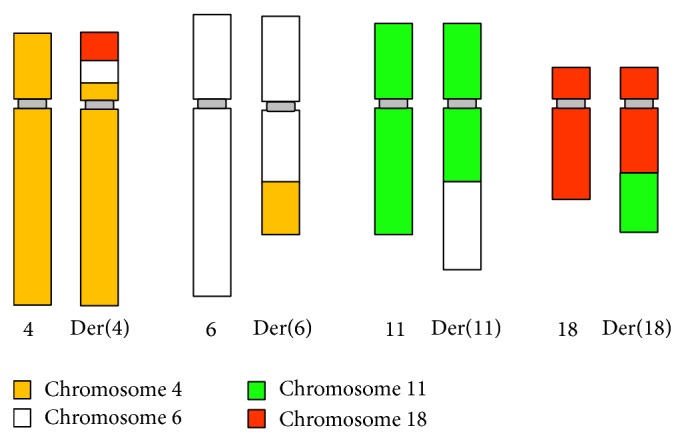
Cartoon summary of the BCCR carried by the proband's mother and grandmother.

To confirm the BCCR karyotyping result, we performed whole chromosome painting FISH (WCP FISH) (Cytocell Technologies Ltd., Cambridge, UK) using probes for chromosomes 4, 6, 11, and 18 on chromosome spreads from the mother and grandmother of the proband, using standard techniques. The hybridisation patterns were consistent with the karyotyping results (Figure 3).  Figure 4 shows a cartoon summary of the BCCR based on the karyotype and whole chromosome painting FISH results.

To investigate whether the BCCR was balanced at a molecular level, we analysed DNA from the proband's mother using Affymetrix CytoScan HD microarray (Affymetrix, Santa Clara, CA, USA), with interpretation based on the NCBI36/hg18 (March 2006) human reference sequence. The microarray result showed no clinically significant genomic imbalance. In particular, there was no evidence for copy number imbalance on chromosomes 4, 6, 11, and 18 in the regions of the translocation breakpoints. Therefore, the CCR appeared to be balanced at the effective resolution of this array (approximately 25–50 kb).

## 3. Discussion

Complex chromosome rearrangements like the one segregating in this family are categorized as exceptional CCRs, which are the most complicated form of CCRs. This complexity results in a greater potential for producing unbalanced gametes during meiosis. A successful pregnancy is rare because the BCCR carrier has a risk for an abnormal conception due to either malsegregation of derivative chromosomes or generation of recombinant chromosomes [1]. According to Gorski et al. [8], there are 4 possible pregnancy outcomes for the BCCR carrier; these are abortion, a liveborn infant with unbalanced chromosomes, an infant carrying the BCCR, or a liveborn infant with normal chromosomes. In the familial exceptional CCR cases described here, we found one case of normal phenotype associated with de novo BCCR (maternal grandmother), one case of normal phenotype associated with maternal inheritance of the BCCR (mother), and one case of abnormal phenotype due to maternal inheritance of an unbalanced form of the CCR (proband). Several familial CCRs reported previously described mostly unbalanced karyotypes [12–15]. In addition, the proband's aunty has inherited normal chromosomes from her BCCR carrier mother. Her normal karyotype result is consistent with her unremarkable reproductive history of 5 phenotypically normal sons and no miscarriages (Figure 1).

WCP FISH was performed to confirm the chromosome rearrangement identified by conventional karyotyping. WCP FISH is a highly sensitive and specific technique that can be used to identify both numerical and structural chromosomal aberrations [16, 17], as well as cryptic genomic imbalances from individuals with apparently balanced chromosomal rearrangements [18, 19]. This technique is a very useful and necessary tool to help characterise the CCR [18–22]. As shown in Figure 3, the WCP FISH supported the karyotype findings and helped confirm the insertional rearrangement of a segment of chromosome 6q into the short arm of chromosome 4, lending support to this CCR being balanced at the cytogenetic level in the proband's mother and grandmother.

Whole genome microarrays have the ability to detect unbalanced de novo and inherited chromosomal abnormalities smaller than 3–5 Mb in size, which is below the resolution of conventional karyotyping [18, 19, 23, 24]. In this study, microarray analysis was used to confirm the apparently balanced nature of the CCR carried by the proband's mother. No evidence for copy number imbalance was observed around the CCR breakpoints, or elsewhere in the genome, which excludes the presence of smaller, cryptic imbalances at the resolution of this array. This analysis supports the CCR being balanced at a molecular level and is consistent with the normal phenotype of BCCR carriers in this pedigree. A repeat sample from the proband was not available for microarray analysis, which would have been helpful to more accurately characterise the CCR breakpoints in the derivative chromosome 4. Microarray analysis has become a valuable tool for investigating apparently balanced chromosomal rearrangements associated with abnormal phenotype, where cryptic deletions below the resolution of light microscopy are often detected around the translocation breakpoints [18, 19]. Unbalanced rearrangements can also be characterised more fully at the molecular level.

A minimum of 70 unbalanced gametes are theoretically possible due to 4:4, 5:3, 6:2, and 7:1 segregations from an octavalent in the case of a CCR involving five breakpoints in four chromosomes [1]. The proband's abnormal karyotype is the result of a 4:4 unbalanced segregation, which is responsible for her abnormal phenotype. The derivative chromosome 4 harbours two additional segments that involve an insertion from chromosome 6q and a translocation from chromosome 18q, which leads to partial trisomy for these chromosomes, in addition to a partial monosomy of 4p. This derivative chromosome is the result of meiotic events producing abnormal gametes [25]. The risk of abnormal offspring from BCCR carriers in this family is high due to the large number of chromosomes and breakpoints involved. Based on a calculation of 4:4 segregation, the possibility of inheriting the balanced form of this CCR is only 2.8% given random segregation of octavalent chromosomes. This is supported by several previous reports [8, 26]. Gorski et al. [8] reported that the higher the number of chromosomal breakpoints and the more complex the chromosomal rearrangement, the higher the ratio of gametes with abnormal chromosomes.

Familial transmission of the BCCR in our pedigree was through female carriers. This is supported by previous reports that record familial transmissions occurring mainly through female carriers [4, 6, 21]. Gardner and Sutherland [1] and Pellestor et al. [4] reported that BCCR in males can cause subfertility and sterility due to disturbances of gametogenesis, whereas in females gametogenesis can escape from this complexity. Thus, females with BCCR can be fertile, have pregnancies, and potentially deliver normal children. This is consistent with the evidence from our pedigree, which includes two healthy female carriers in whom the rearrangements are balanced and another who has inherited normal chromosomes from a BCCR carrier female.

Due to the complex nature of BCCRs, genetic counselling will always be difficult. The reproductive risk is very specific for each carrier, and the precise risk may be impossible to establish [9]. Reproductive histories may also vary between carriers of the same BCCR, as is evidenced in our own pedigree, where the proband's mother has a poorer reproductive history than the proband's grandmother.

In conclusion, the proband's abnormal clinical presentation was caused by inheriting a derivative chromosome 4, which is an unbalanced form of the BCCR carried by her mother. Although the possibility of having a normal child is greatly reduced, a daughter has inherited the CCR in its balanced form, and another daughter has inherited normal chromosomes. This case is instructive in demonstrating that an exceptional BCCR can be inherited in its full balanced form to the next generation.
